# Unveiling the Genetic Diversity and Population Structure of the Endangered Fern *Angiopteris fokiensis* Through Genome Survey and Genomic SSR Markers

**DOI:** 10.3390/biom15121649

**Published:** 2025-11-24

**Authors:** Yiwei Zhou, Jianjun Tan, Lishan Huang, Yanyu Luo, Shaoli Huang, Yuanjun Ye, Yechun Xu

**Affiliations:** 1 Guangdong Provincial Key Laboratory of Ornamental Plant Germplasm Innovation and Utilization, Environmental Horticulture Research Institute, Guangdong Academy of Agricultural Sciences, Guangzhou 510640, China; zhouyiwei6333@163.com (Y.Z.);; 2 Guangzhou Academy of Agricultural and Rural Sciences, Bureau of Agriculture and Rural Affairs of Guangzhou Municipality, Guangzhou 510335, China

**Keywords:** *Angiopteris fokiensis*, conservation genetics, genomic SSRs, genetic diversity, endangered fern, population structure

## Abstract

*Angiopteris fokiensis* is an endangered fern with ecological and medicinal value, yet genetic studies to support its conservation have been scarce. We performed a genome survey using high-throughput sequencing, developed genomic SSR markers from a draft assembly, and genotyped 96 individuals from 10 populations in Guangdong Province. The genome size was ~4.44 Gb (1.89% heterozygosity). From a 3.58 Gb contig assembly, 4,327,181 SSR loci were identified, with 15 highly polymorphic SSR markers being developed. Genotyping showed high within-population genetic diversity, low inter-population differentiation, and 98.55% of variation within populations. Bayesian structure, principal coordinates analysis, and neighbor-joining tree analyses consistently indicated admixed genetic clusters without clear geographical division. Additionally, the analysis revealed no significant correlation between genetic and geographic distances. Conservation should prioritize intra-population diversity via in situ/ex situ strategies. This study provides the first genomic SSR resources for *A. fokiensis* and underscores the importance of conserving within-population genetic diversity through integrated in situ and ex situ strategies.

## 1. Introduction

The genus *Angiopteris* Hoffmann, belonging to the family Marattiaceae, comprises perennial herbaceous plants. It includes approximately 50 species worldwide, among which 30 occur in China, including 17 endemic species [1]. Members of this genus exhibit a chromosome base number of n = 40, with many species, such as *Angiopteris lygodiifolia* and *Angiopteris palmiformis*, identified as diploids (2n = 80) [2,3]. In 2021, the National Forestry and Grassland Administration and the Ministry of Agriculture and Rural Affairs of the People’s Republic of China jointly issued the updated List of National Key Protected Wild Plants, which includes all species of *Angiopteris* (https://www.gov.cn/zhengce/zhengceku/2021-09/09/content_5636409.htm, accessed on 1 September 2021). Plants of this genus possess considerable ornamental and medicinal value. Current research on *Angiopteris* primarily focuses on species surveys [4], phylogenetic analyses [5,6,7], plastid genome assembly [8,9], chemical composition [10,11,12], and medicinal applications [13,14,15,16]. Moreover, new species such as *Angiopteris sugongii* [17] and *Angiopteris nodosipetiolata* [18] continue to be discovered. Despite its protected status, studies on population differentiation within specific species across regions remain limited, hindering the formulation of effective conservation strategies.

*Angiopteris fokiensis* Hieron., native to southern China, is predominantly distributed in Guangdong, Guangxi, Fujian, and other provinces. Since the beginning of the 21st century, the high ornamental value of *A. fokiensishas* led to extensive illegal harvesting, resulting in a sharp decline in its population and pushing it to the brink of extinction. Although the species was once widely distributed across southern China, it has now been listed as a Class II nationally protected wild plant due to its significant ecological importance. Recent studies by Chinese researchers have investigated the chemical constituents of its roots and leaves [19,20], as well as its resource distribution in certain nature reserves [21]. However, research on population genetic differentiation across different regions remains scarce. In particular, the lack of effective molecular markers for assessing genetic diversity among populations impedes the development of appropriate conservation strategies for this endangered species.

Simple sequence repeats (SSRs) are widely distributed throughout the genomes of eukaryotes. Owing to their high information content, co-dominant inheritance, pronounced polymorphism, and reproducibility, SSRs are among the most widely used markers for plant genotyping [22]. Next-generation sequencing enables efficient development of species-specific SSR markers [23]. Several non-model plants, such as *Hedychium* [24] and *Curcuma alismatifolia* [25], have undergone genetic diversity assessments using genomic SSRs developed from genome sequences. To date, there have been no reports on the development of molecular markers or genetic diversity studies for *Angiopteris fokiensis*. The development of genomic SSR markers for *A. fokiensis* would facilitate research on its population genetics and provide critical insights for the conservation of this species.

In this study, we collected samples from ten populations of *A. fokiensis* across different locations in Guangdong Province, China. Using high-throughput sequencing, we sequenced and preliminarily assembled the genome of *A. fokiensis*. Based on the assembled sequences, we developed genomic SSR (g-SSR) markers and evaluated the genetic diversity of these ten populations. This work provides valuable molecular tools and informed strategies for the conservation of *A. fokiensis*.

## 2. Materials and Methods

### 2.1. Sampling of Plant Materials

**Figure 1:**
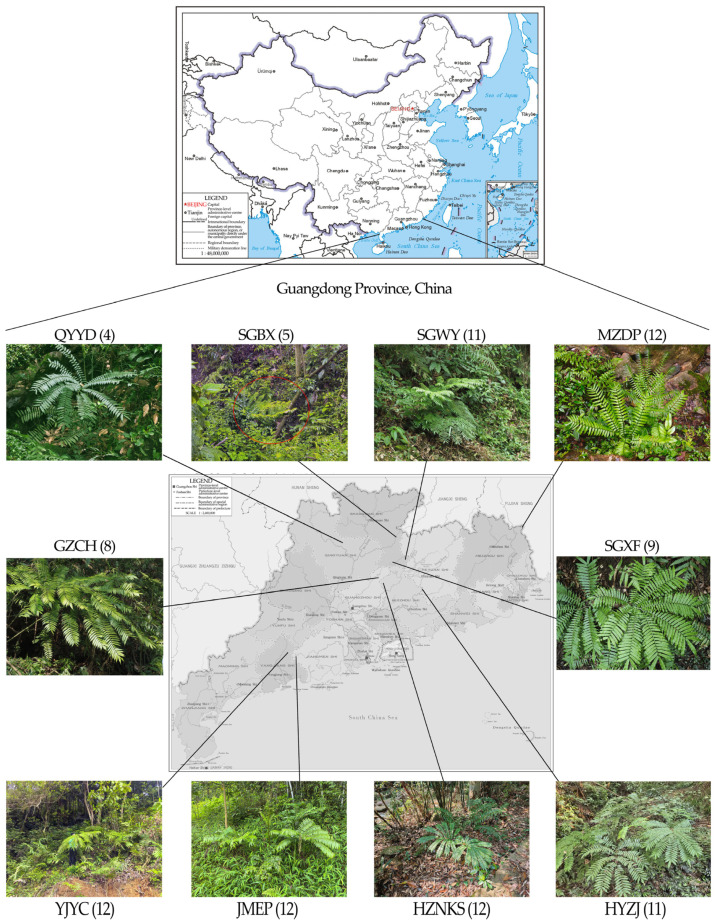
Locations of 10 wild populations of *A. fokiensis* sampled in Guangdong Province, China. The numbers associated with each population indicate the corresponding sample size. The plant circled in red is *A. fokiensis*.

A total of 96 young leaf samples of *A*. *fokiensis* were collected from ten locations in Guangdong Province, China (Figure 1). Sampling sites included: Conghua District, Guangzhou (GZCH; E 113.8085, N 23.7197); Yingde City, Qingyuan (QYYD; E 113.0789, N 24.4037); Qingyunshan Provincial Nature Reserve, Wengyuan County, Shaoguan (SGWY; E 114.2598, N 24.2928); Yunjishan Provincial Nature Reserve, Xinfeng County, Shaoguan (SGXF; E 114.1742, N 24.0911); Banxi Municipal Nature Reserve, Wengyuan County, Shaoguan (SGBX; E 114.1324, N 24.357); Nankun Mountain, Longmen County, Huizhou (HZNKS; E 113.8305, N 23.6364); Dongjiang Forest Farm, Zijin County, Heyuan (HYZJ; E 114.7104, N 23.4352); Qixingkeng Provincial Nature Reserve, Enping City, Jiangmen (JMEP; E 112.1039, N 22.1864); Fengxi Forest Farm, Dapu County, Meizhou (MZDP; E 116.7819, N 24.6458); and Yangchun City, Yangjiang (YJYC; E 111.9943, N 22.3218). The number of individuals collected from each site was as follows: GZCH: 8 individuals, QYYD: 4 individuals, SGWY: 11 individuals, SGBX: 5 individuals, SGXF: 9 individuals, HZNKS: 12 individuals, HYZJ: 11 individuals, JMEP: 12 individuals, MZDP: 12 individuals, and YJYC: 12 individuals.

### 2.2. Genome Sequencing and Preliminary Assembly

Genomic DNA was extracted from leaf tissues using the Plant Genomic DNA Kit (DP305; Tiangen Biotech, Beijing, China). Qualified DNA samples were randomly sheared into fragments of approximately 350 bp using a Covaris ultrasonicator. Sequencing libraries were constructed through end repair, A-tailing, adapter ligation, bead-based purification, and PCR amplification. Paired-end (150 bp) sequencing was performed on the MGI T7 platform. Raw reads were processed with fastp v0.23.0 [26] using parameters “-q 19 -u 50 -n 5” to remove adapter-contaminated sequences (>5 bp), reads with >50% bases having Q ≤ 19, and sequences with >5% N bases. Genome characteristics were evaluated using a two-step approach: K-mer frequency distribution was analyzed with Jellyfish v2.2.10 [27] (-m 21), and results were input into GenomeScope 2.0 [28] (-k 21) to estimate genome size, heterozygosity, and repeat content. A preliminary genome assembly was generated with Minia v3.2.4 [29] using a k-mer size of 35 and 20 cores. Assembly quality was assessed with QUAST v5.2.0 [30] based on contig number, N50, and genome coverage.

### 2.3. Genome-Wide SSR Identification and Primer Design

SSRs across the genome of *A. fokiensis* were detected using the MISA tool [31], with searches conducted on both chloroplast genome and unigene sequences. The following criteria were applied for SSR recognition: a minimum of 10 repeats for mononucleotide motifs, 6 repeats for dinucleotides, and 5 repeats for tri- to hexanucleotide SSR motifs. Corresponding primers were designed with the online platform Primer 3.0 [32].

### 2.4. SSR-PCR Analysis

Following quality verification via agarose gel electrophoresis (Figure S1), genomic DNA from the 96 accessions was diluted to approximately 50 ng/μL for SSR-PCR. All 196 SSR primer pairs were commercially synthesized by Sangon Biotech (Shanghai, China). Initial screening was carried out using four individuals representing distinct populations, from which 17 primers exhibiting clear polymorphism and high reproducibility were chosen for subsequent population genetics analysis (Table S1). Fluorescence-based (FAM, HEX, ROX and TAMRA) PCR was conducted according to an established protocol [33] under the following thermal cycling conditions: initial denaturation at 94 °C for 5 min; 30 cycles of 94 °C for 30 s, 58 °C for 30 s, and 72 °C for 1 min; followed by 13 cycles of 94 °C for 30 s, 53 °C for 30 s, and 72 °C for 1 min; with a final extension at 72 °C for 10 min. The amplified fragments were separated and sized via capillary electrophoresis using an ABI 373xl Genetic Analyzer (Applied Biosystems, Foster City, CA, USA).

### 2.5. Genetic Diversity Analysis

The software Micro-Checker v2.2 [34] was used to screen for null alleles. Loci with a high frequency of null alleles were excluded, following the criterion established by previous research [35]. The statistical power was assessed using G*Power 3.1 software (https://www.psychologie.hhu.de/arbeitsgruppen/allgemeine-psychologie-und-arbeitspsychologie/gpower, accessed on 10 November 2025). Genetic diversity parameters, including the number of alleles (*Na*), effective number of alleles (*Ne*), Shannon’s information index (*I*), observed heterozygosity (*Ho*), and expected heterozygosity (*He*), an individual relative to the subpopulation (*Fis*), the inbreeding coefficient of an individual relative to the total population (*F_it_*), the genetic differentiation among subpopulations (*F_st_*), and a Mantel test (correlating genetic and geographic distances), were calculated using GenAlEx 6.5 [36]. Linkage disequilibrium (LD) analysis among loci was performed using TASSEL 4.0 software [37], which computed the *R*^2^ and *D*′ values between loci. Analysis of molecular variance (AMOVA) and principal coordinates analysis (PCoA) were also performed. Polymorphic information content (*PIC*) was computed with Powermarker 3.25 [38]. Nei’s genetic distance was used to construct a neighbor-joining (NJ) tree, which was visualized in MEGA 7 [39]. Population structure was inferred using a Bayesian clustering approach in STRUCTURE 2.3.4 [40] with the admixture model. Ten independent runs were performed for each *K* value (1–12). The optimal *K* value was determined using Structure Harvester [41], and results were integrated with CLUMPP v1.1.2 [42]. A general linear model was applied in R to assess the correlation between geographic and genetic distances.

## 3. Results

### 3.1. Genome Survey and Preliminary Assembly

Raw sequencing data generated from the MGI T7 platform underwent stringent quality control to remove paired-end reads shorter than 100 bp, low-quality sequences, and adapter-contaminated reads. This process yielded a total of 3,425,037,788 high-quality reads, representing 513,755,668,200 bp (Table S2). Quality assessment showed that 97.12% of bases had a base call accuracy exceeding 99.9% (Q_30_). Based on K-mer frequency analysis using GenomeScope 2.0, the maximum estimated genome size was 4,441,672,626 bp, with a heterozygosity rate of 1.89% and a model fit of 95.49% (Table S3). The minimum estimated genome size was 4,427,241,777 bp, with a heterozygosity of 1.85% and a model fit of 42.12% (Table S3). A preliminary de novo assembly produced contigs totaling 3,584,469,236 bp, comprising 45,054,615 contigs (Table S4). The longest contig was 21,998 bp, and 660,646 contigs ranged between 300 bp and 500 bp. The overall GC content was 39.67%.

### 3.2. SSR Mining in the Genome of Angiopteris fokiensis

From the 45,054,615 contigs, a total of 4,327,181 SSR markers belonging to 291 motif types were identified (Table S5). Among these, 580,088 contigs contained more than one SSR. Dinucleotide repeats were the most abundant (3,508,767; 81.09%), followed by mononucleotide (520,015; 12.02%), tetranucleotide (219,073; 5.06%), trinucleotide (71,771; 1.66%), hexanucleotide (4596; 0.11%), and pentanucleotide repeats (2959; 0.07%).

**Figure 2:**
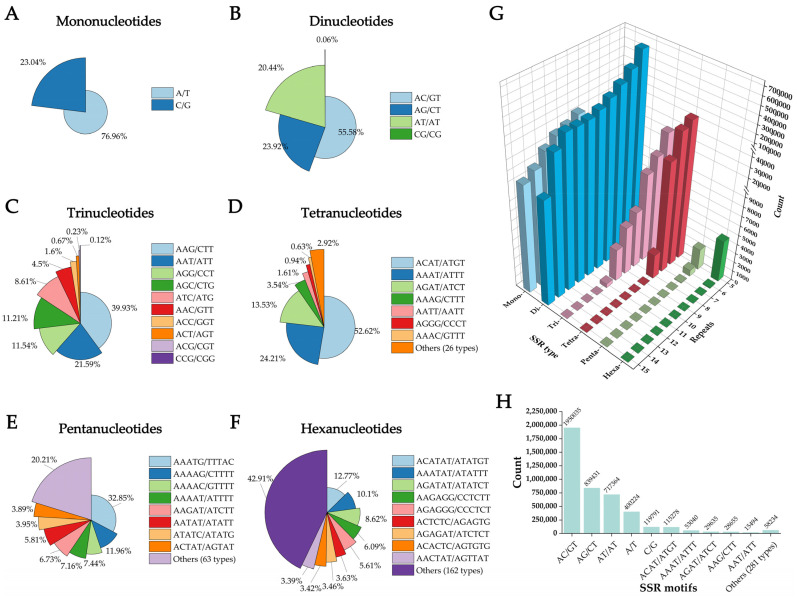
Mining of SSR loci in the *A. fokiensis* genome. (**A**–**F**) Frequency distribution of SSR motifs by type: (**A**) Mononucleotides, (**B**) Dinucleotides, (**C**) Trinucleotides, (**D**) Tetranucleotides, (**E**) Pentanucleotides, (**F**) Hexanucleotides. (**G**) Count of different repeat times for the six motif types. (**H**) Frequency distribution of the dominant SSR motifs.

Mononucleotide repeats were predominantly composed of A/T (76.96% of the total), in contrast to C/G repeats which constituted only 23.04% (Table S6). Among dinucleotide repeats, AC/GT was most frequent (55.58%), followed by AG/CT (23.92%) and AT/AT (20.44%), while CG/CG was rare (0.06%) (Figure 2). For trinucleotide repeats, AAG/CTT was predominant (39.93%), with AAT/ATT (21.59%), AGG/CCT (11.54%), and AGC/CTG (11.21%) also common. Tetranucleotide repeats were dominated by ACAT/ATGT (52.62%), followed by AAAT/ATTT (24.21%) and AGAT/ATCT (13.53%). Pentanucleotide repeats were led by AAATG/CATTT (32.85%), and the most frequent hexanucleotide motifs included ACATAT/ATATGT (12.77%) and AAATAT/ATATTT (10.10%). The abundance of SSRs generally decreased with increasing repeat length, with repeat numbers of 6, 7, and 10 being most common (Figure 2). Ten SSR motifs occurred more than 10,000 times, reflecting the dominant SSR types in *A. fokiensis* (Figure 2).

### 3.3. Population Genetic Diversity Based on Polymorphic SSR Markers

To evaluate genetic diversity, 17 highly polymorphic and reproducible SSR markers were selected from an initial set of 196 and used to genotype 10 populations from Guangdong Province, China. Based on the null allele screening results from Micro-Checker v2.2, two loci (AfgSSR-119 and AfgSSR-168) with a high null allele frequency were excluded (Table S7). The remaining 15 high-quality loci were retained for subsequent analyses. An a priori analysis conducted using G*Power 3.1 indicated low statistical power, implying a limited ability to detect weak genetic effects in this study; therefore, the following results should be interpreted as preliminary findings under this power constraint. Linkage disequilibrium (LD) analysis revealed generally low *R*^2^ and *D*′ values among the 15 SSR loci. The highest *R*^2^ value (0.41) was observed between AfgSSR-55 and AfgSSR-145, although their *D*′ value was only 0.36. In contrast, AfgSSR-47 and AfgSSR-3 showed the highest *D*′ value, but their *R*^2^ was only 0.03. The overall low values of *D*′ and *R*^2^ indicate a lack of strong linkage disequilibrium and suggest that these loci are inherited independently (Figure S2).

The 15 selected markers displayed varying levels of polymorphism. The number of alleles (*Na*) per locus ranged from 1.400 (AfgSSR-35) to 9.700 (AfgSSR-55), with a mean of 3.573 (Table 1). The effective number of alleles (*Ne*) varied between 1.061 (AfgSSR-35) and 7.439 (AfgSSR-55), averaging 2.385. Shannon’s information index (*I*) values spanned from 0.101 (AfgSSR-35) to 2.096 (AfgSSR-55), with a mean of 0.837. Observed heterozygosity (*Ho*) ranged from 0.022 (AfgSSR-35) to 0.988 (AfgSSR-3), averaging 0.441. Expected heterozygosity (*He*) and polymorphic information content (*PIC*) also varied across markers, with mean values of 0.445 and 0.441, respectively. Markers such as AfgSSR-55 showed high polymorphism, whereas AfgSSR-35 exhibited low diversity.

### 3.4. Population Genetic Differentiation

Subsequent population-level analyses revealed further genetic patterns. Genetic diversity parameters for each population were calculated using GenAlEx 6.5. MZDP had the highest number of alleles (*Na* = 4.067), while QYYD had the lowest (2.533) (Table 2). The effective number of alleles (*Ne*) was highest in JMEP (2.713) and lowest in QYYD (2.003). Shannon’s index was highest in MZDP (0.926) and lowest in QYYD (0.666). Observed heterozygosity (*Ho*) was highest in SGXF (0.550) and lowest in HZNKS (0.396). Expected heterozygosity (*He*) was greatest in MZDP (0.482) and lowest in QYYD (0.381).

**Figure 3:**
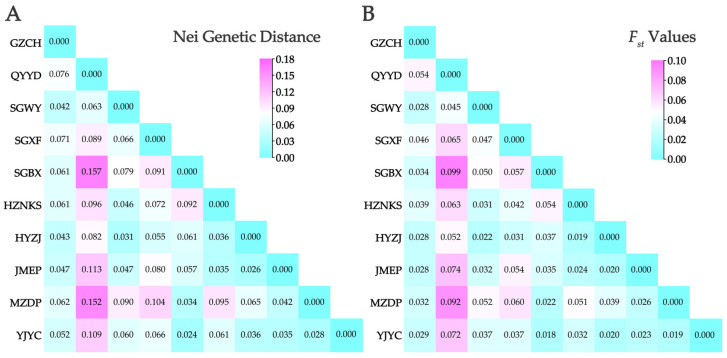
Genetic relationships among 10 *A. fokiensis* populations based on 15 gSSR markers: (**A**) Nei’s genetic distance; (**B**) Pairwise *F_st_* values.

To quantify inbreeding, genetic diversity, and population structure, the inbreeding coefficients (*F_is_* and *F_it_*), and population differentiation (*F_st_*) were analyzed (Table S8). Six loci exhibited negative *F_is_* values, a pattern potentially indicative of outbreeding or population substructure. In contrast, nine loci showed positive *F_is_* values, which may suggest heterozygote deficiency. *F_it_* values followed trends similar to *F_is_* but differed in magnitude, reflecting additional population-level effects. High *F_it_* values (e.g., 0.946 for AfgSSR-119) indicated strong heterozygote deficiency. *F_st_* values ranged from 0.035 to 0.127 (mean = 0.073), indicating low genetic differentiation among subpopulations. Consistent with this, Nei’s genetic distance (0.025–0.162) and *F_st_* (0.017–0.096) between population pairs were low, with the smallest differentiation between SGBX and YJYC and the largest between QYYD and SGBX (Figure 3).

### 3.5. NJ Clustering, PCoA, and Genetic Structure Analysis

**Figure 4:**
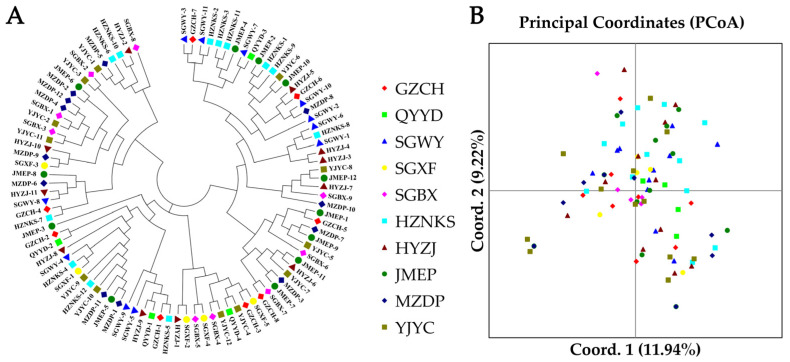
Analysis of 96 *A. fokiensis* accessions from 10 populations based on 15 gSSR markers: (**A**) Neighbor-joining (NJ) dendrogram; (**B**) PCoA score plot.

**Figure 5:**
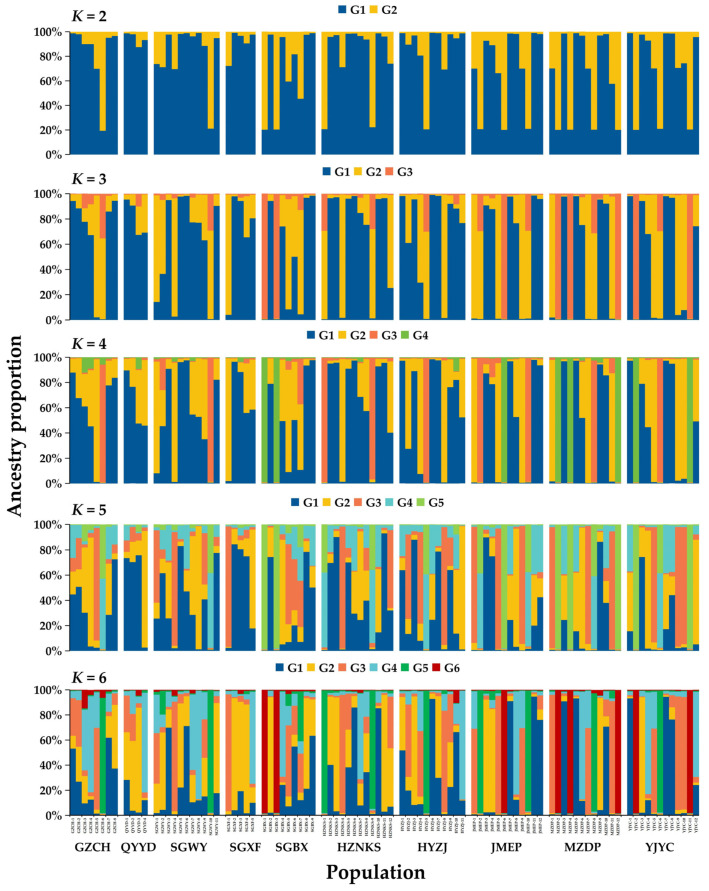
Population genetic structure of 96 *A. fokiensis* accessions from 10 populations inferred from 15 gSSR markers.

To visualize genetic relationships, NJ trees and PCoA were constructed. Both analyses showed extensive intermixing of individuals across the 10 populations, with no clear geographic clustering (Figure 4). Bayesian population structure analysis indicated no homogeneous genetic clusters across *K* = 2–6. The most supported structure (*K* = 4, based on Delta *K*) revealed four ancestral genetic components, with all populations showing mixed ancestry (Table S9). Higher *K* values further resolved substructure but consistently highlighted genetic admixture and the absence of discrete population divergence (Figure 5).

### 3.6. AMOVA and Correlation Between Genetic and Geographic Distances

**Figure 6:**
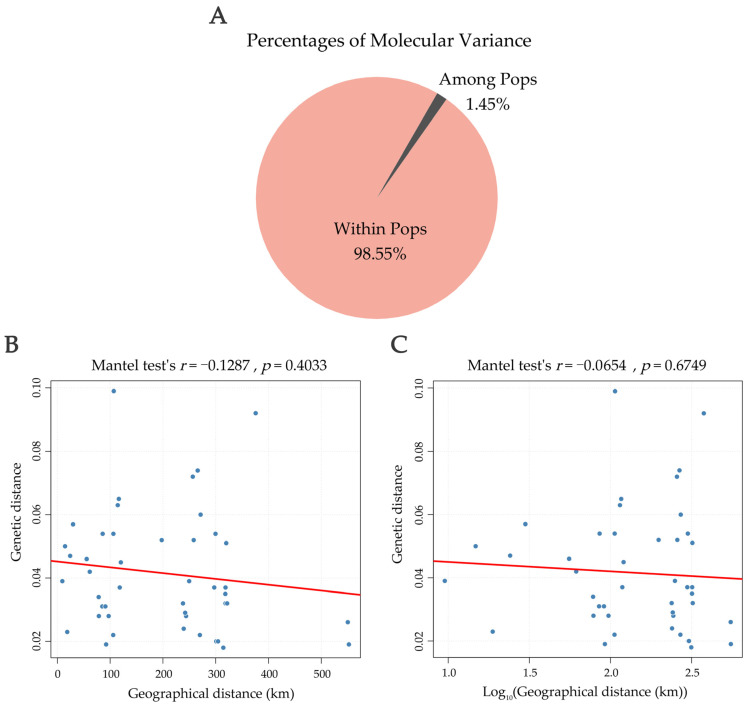
Analysis of Molecular Variance (AMOVA) and the relationship between genetic and geographical distances. (**A**) Distribution of molecular variance components. (**B**,**C**) Results of the Mantel test assessing the correlation between genetic distance and (**B**) raw geographical distance, and (**C**) Log10-transformed geographical distance. The red line indicates the fitted regression line.

Analysis of molecular variance (AMOVA) indicated that only 1.45% of total genetic variation occurred among populations, while 98.55% resided within populations, underscoring the high level of intra-population diversity (Figure 6; Table S10). Furthermore, a Mantel test revealed no significant correlation between genetic and geographic distances (*r* = −0.1287, *p* = 0.4033), nor between genetic distance and Log10-transformed geographic distance (*r* = −0.0654, *p* = 0.6749), reinforcing that geographic isolation has not been a major factor shaping genetic structure in *A. fokiensis*.

## 4. Discussion

Ferns are recognized for their exceptionally large genomes [43]. Several fern species with sequenced genomes include *Dipteris shenzhenensis* (1.9 Gb) [44], *Cibotium barometz* (3.49 Gb) [45], *Adiantum capillus*-*veneris* (4.83 Gb) [46], *Alsophila spinulosa* (6.23 Gb) [47], *Adiantum nelumboides* (6.27 Gb) [48], *Ceratopteris richardii* (7.46 Gb) [43], and *Marsilea vestita* (10.40 Gb) [49]. In this study, genome survey analysis estimated the genome size of *A. fokiensis* to be approximately 4.43–4.44 Gb, placing it in the medium-to-large size range among ferns. A preliminary assembly yielded contigs totaling 3.58 Gb. These results expand genomic knowledge of ferns, particularly for the genus *Angiopteris* within the Marattiaceae family. Future work should aim to generate a chromosome-level genome assembly for *A. fokiensis* by integrating long-read sequencing technologies such as HiFi, which will facilitate further investigation of population differentiation.

Information on genetic diversity is essential for developing conservation strategies for endangered species [50,51]. SSR markers have been widely used to assess genetic diversity in many threatened plants, such as *Picea neoveitchii* [52], *Tetracentron sinense* [53], *Hemsleya zhejiangensis* [54], and *Taxus contorta* [55]. In this study, a total of 4,327,181 SSR loci were identified from the assembled contigs of *A. fokiensis*, of which 15 polymorphic genomic SSRs were developed and used to genotype 10 populations. The mean *PIC* was 0.441, indicating that these markers are effective for evaluating population genetic diversity. Five markers—AfgSSR-3, AfgSSR-21, AfgSSR-55, AfgSSR-95, and AfgSSR-139—showed *PIC* values greater than 0.5, suggesting high informativeness. Given the transferability of SSR markers across related taxa, the polymorphic g-SSRs developed here may also be applicable to genetic diversity studies in other *Angiopteris* species. The 10 sampled populations exhibited relatively high genetic diversity, with mean Shannon’s index (*I*) of 0.837 and expected heterozygosity (*He*) of 0.445. High genetic diversity is beneficial for species adaptation to environmental changes. Previous studies have indicated that outcrossing species generally maintain higher genetic diversity and lower population differentiation than selfing species [56], as observed in *Saussurea involucrata* [57]. Thus, the outcrossing breeding system of *A. fokiensis* is likely a key factor in maintaining its high genetic diversity.

Moderate-to-high gene flow among populations is crucial for preventing inbreeding depression and conserving genetic variation [58]. In eight of the 10 populations, observed heterozygosity (*Ho*) was lower than expected heterozygosity (*He*), indicating a relative excess of homozygotes and suggesting possible inbreeding or increased clonal reproduction through rhizome propagation. However, the low average *F_st_* value indicate substantial genetic exchange and limited differentiation among the *A. fokiensis* populations in Guangdong. This was supported by AMOVA, which revealed that most genetic variation occurred within populations (98.46%), with only 1.54% among populations. However, due to the low statistical power of the study, the *F_st_* value should be interpreted as a preliminary indicator that provides ancillary support for the conclusion of weak population differentiation. Moreover, the small sample sizes of certain populations—such as QTTD and SGFX, represented by only four and five individuals, respectively—may have introduced considerable bias in the estimation of population parameters (e.g., *He* and *Ho*). Consequently, these findings should be regarded as exploratory. Future studies should increase the number of individuals sampled per population to improve estimation accuracy. Thus, we emphasize that the primary contribution of this work lies in providing baseline data and initial insights to inform subsequent research. Regarding the potential for clonality, the observed heterozygote deficit (*Ho* < *He*) could be consistent with partial clonal reproduction through rhizome propagation. However, the relatively high genetic diversity observed across populations suggests that sexual reproduction remains a significant contributor to population maintenance. Without direct genotypic analysis to identify clones, we cannot definitively confirm or exclude the occurrence of clonality, but our data indicate that both reproductive modes may coexist in *A. fokiensis*.

Cluster analysis, PCoA, and population structure analysis consistently showed minimal genetic differentiation among populations. Similar genetic patterns have been reported in other outcrossing endangered plants such as *Saussurea involucrata* [57] and *Pulsatilla patens* [59]. Surprisingly, although this species is listed as a national Category II protected plant due to its endangered status, our analyses revealed relatively high genetic diversity within the sampled populations. This finding appears counterintuitive from a purely biological perspective. One plausible explanation is the substantial impact of anthropogenic pressures; specifically, intensive illegal harvesting in a short period may have caused a rapid decline in its wild population size. Many plant species face extinction due to ecological threats—in this case, severe human disturbance—before genetic factors exert significant effects [60]. This view is indirectly supported by the absence of a significant correlation between geographic and genetic distances. Nevertheless, the underlying mechanisms require further validation through long-term monitoring and expanded population surveys.

A major limitation of this study is the small sample size from each population, a consequence of the species’ critically endangered status, which may affect the accuracy of inferences regarding the causes of its decline. Furthermore, genetic studies of *A. fokiensis* have long been hindered by the lack of genomic data and molecular markers. This study represents an initial effort to address these gaps, including a preliminary genome assembly and the development of 15 polymorphic SSR markers, which provide a foundation for basic genetic diversity assessments. In future work, we will expand field sampling to obtain a larger set of specimens that meet the statistical requirements for robust population genetic analysis. We also aim to generate a chromosome-scale genome assembly for *A. fokiensis* by integrating Hi-C and long-read sequencing technologies. Genome-wide markers will then be used to comprehensively investigate the genetic structure and diversity of wild populations, offering insights for the conservation biology of *A. fokiensis* and related species.

This study employed genomic SSR markers to analyze genetic variation within and among 10 populations of *A. fokiensis* in Guangdong Province, providing insights useful for conservation planning. Due to its ornamental and medicinal value, *A. fokiensis* has experienced overharvesting, leading to population decline. In response, all *Angiopteris* species were included in the List of National Key Protected Wild Plants issued by the National Forestry and Grassland Administration and the Ministry of Agriculture and Rural Affairs of China in 2021. Our results indicate limited genetic differentiation among the studied populations. Therefore, conservation strategies should prioritize maintaining genetic diversity within populations—for instance, by minimizing inbreeding depression and preserving key individuals—rather than focusing on specific populations. A combined approach of in situ conservation (e.g., establishing nature reserves) and ex situ measures (e.g., cultivation and reintroduction) is recommended to preserve genetic representation. In addition to spore-based reproduction, rhizomatous clonal propagation is another means of population maintenance in *A. fokiensis*. If clonal growth becomes dominant in fragmented or disturbed habitats, it may accelerate genetic homogenization and reduce adaptive potential. Therefore, continuous monitoring of reproductive mode and genetic diversity in protected populations is strongly advised.

## 5. Conclusions

This study presents the first comprehensive assessment of genetic diversity and population structure of *A. fokiensis* using genome-wide SSR markers derived from a contig-level draft genome assembly. Our results demonstrate relatively high genetic diversity and low population differentiation among the surveyed populations in Guangdong, likely facilitated by substantial gene flow and an outcrossing reproductive system. The developed polymorphic g-SSR markers are not only effective for evaluating genetic variation within *A. fokiensis* but also hold promise for related species within the genus. The absence of distinct spatial genetic structure suggests that conservation strategies should emphasize the maintenance of genetic diversity within populations through measures such as reducing inbreeding, protecting habitat connectivity, and implementing complementary ex situ conservation programs. Future studies should incorporate broader geographical sampling and ecological data to better assess the impact of anthropogenic fragmentation on gene flow and adaptive potential. The genomic resources and markers provided herein will facilitate long-term monitoring and support molecular-assisted conservation of this endangered fern.

## Figures and Tables

**Table 1:** Genetic diversity parameters of 15 genomic SSR markers.

SSR Marker	Repeat Motif	*Na*	*Ne*	*I*	*Ho*	*He*	*PIC*
AfgSSR-3	(GA)9	2.900	2.477	0.960	0.988	0.593	0.534
AfgSSR-17	(TC)6	2.200	1.412	0.423	0.055	0.245	0.263
AfgSSR-21	(AAG)9	6.100	4.474	1.603	0.878	0.769	0.794
AfgSSR-35	(AC)8	1.400	1.061	0.101	0.022	0.052	0.059
AfgSSR-47	(CT)6	3.800	1.844	0.807	0.262	0.419	0.446
AfgSSR-55	(AC)9	9.700	7.439	2.096	0.639	0.855	0.898
AfgSSR-59	(AC)8	2.300	1.897	0.691	0.424	0.463	0.397
AfgSSR-93	(CT)7	3.700	2.168	0.911	0.519	0.494	0.494
AfgSSR-95	(GA)9	4.000	2.275	0.973	0.484	0.524	0.512
AfgSSR-131	(TC)6	4.000	1.827	0.863	0.501	0.446	0.448
AfgSSR-139	(TC)7	3.400	2.549	1.011	0.752	0.595	0.609
AfgSSR-141	(CA)8	2.200	1.295	0.359	0.185	0.200	0.221
AfgSSR-145	(CT)6	3.200	2.351	0.940	0.584	0.567	0.482
AfgSSR-185	(TC)6	2.500	1.394	0.449	0.154	0.242	0.232
AfgSSR-189	(AC)6	2.200	1.319	0.371	0.174	0.210	0.223
Mean		3.573	2.385	0.837	0.441	0.445	0.441

Abbreviations: *He*, expected heterozygosity; *Ho*, observed heterozygosity; *I*, Shannon’s index; *Na*, number of alleles; *Ne*, number of effective alleles; *PIC*, polymorphic information content.

**Table 2:** Genetic diversity parameters of 10 wild populations of *A. fokiensis*.

Population	*Na*	*Ne*	*I*	*Ho*	*He*
GZCH	3.333	2.287	0.802	0.417	0.433
QYYD	2.533	2.003	0.666	0.467	0.381
SGWY	3.533	2.270	0.799	0.412	0.425
SGXF	3.067	2.455	0.815	0.550	0.456
SGBX	3.467	2.218	0.811	0.452	0.433
HZNKS	3.933	2.348	0.879	0.396	0.454
HYZJ	3.933	2.661	0.924	0.425	0.479
JMEP	4.000	2.713	0.882	0.430	0.447
MZDP	4.067	2.620	0.926	0.428	0.482
YJYC	3.867	2.278	0.870	0.436	0.459

Abbreviations: *He*, expected heterozygosity; *Ho*, observed heterozygosity; *I*, Shannon’s index; *Na*, number of alleles; *Ne*, number of effective alleles.

## Data Availability

The original contributions presented in this study are included in the Supplementary Materials. Further inquiries can be directed to the corresponding authors.

## Supplementary Materials

The following supporting information can be downloaded at: https://www.mdpi.com/article/10.3390/biom15121649/s1, Figure S1: Agarose gel electrophoresis confirms the quality of DNA extracted from 96 materials. Lane M represents the DNA marker DL2000; Figure S2: Linkage disequilibrium (LD) among 15 loci. Values in the upper and lower triangles represent *R*^2^ and *D*’ measures, respectively; Table S1: Primer sequences of 15 polymorphic SSR markers; Table S2: Summary of genome sequencing data for *A. fokiensis*; Table S3: Genome size estimation and characteristics of *A. fokiensis*; Table S4: Summary of contig statistics and SSR identification from the initial genome assembly of *A. fokiensis*; Table S5: Number of SSR motifs identified in the *A. fokiensis* genome; Table S6: Frequency distribution of SSR numbers for the six motif types; Table S7. Analysis of null allele frequency at 17 loci using Micro-Checker; Table S8: *F*-Statistics over 10 populations for 15 loci; Table S9: Evaluation parameters for population genetic structure analysis; Table S10: Summary of AMOVA result.
