# Supplementary material for: Unveiling the Genetic Diversity and Population Structure of the Endangered Fern Angiopteris fokiensis Through Genome Survey and Genomic SSR Markers
Source: Biomolecules. 2025 Nov 24;15(12):1649. doi: 10.3390/biom15121649 (PMC12730431; doi:10.3390/biom15121649)
Supplement: Supplementary file 1 [file biomolecules-15-01649-s001.zip › Supplementary Figure S1-S2 R2.pdf]

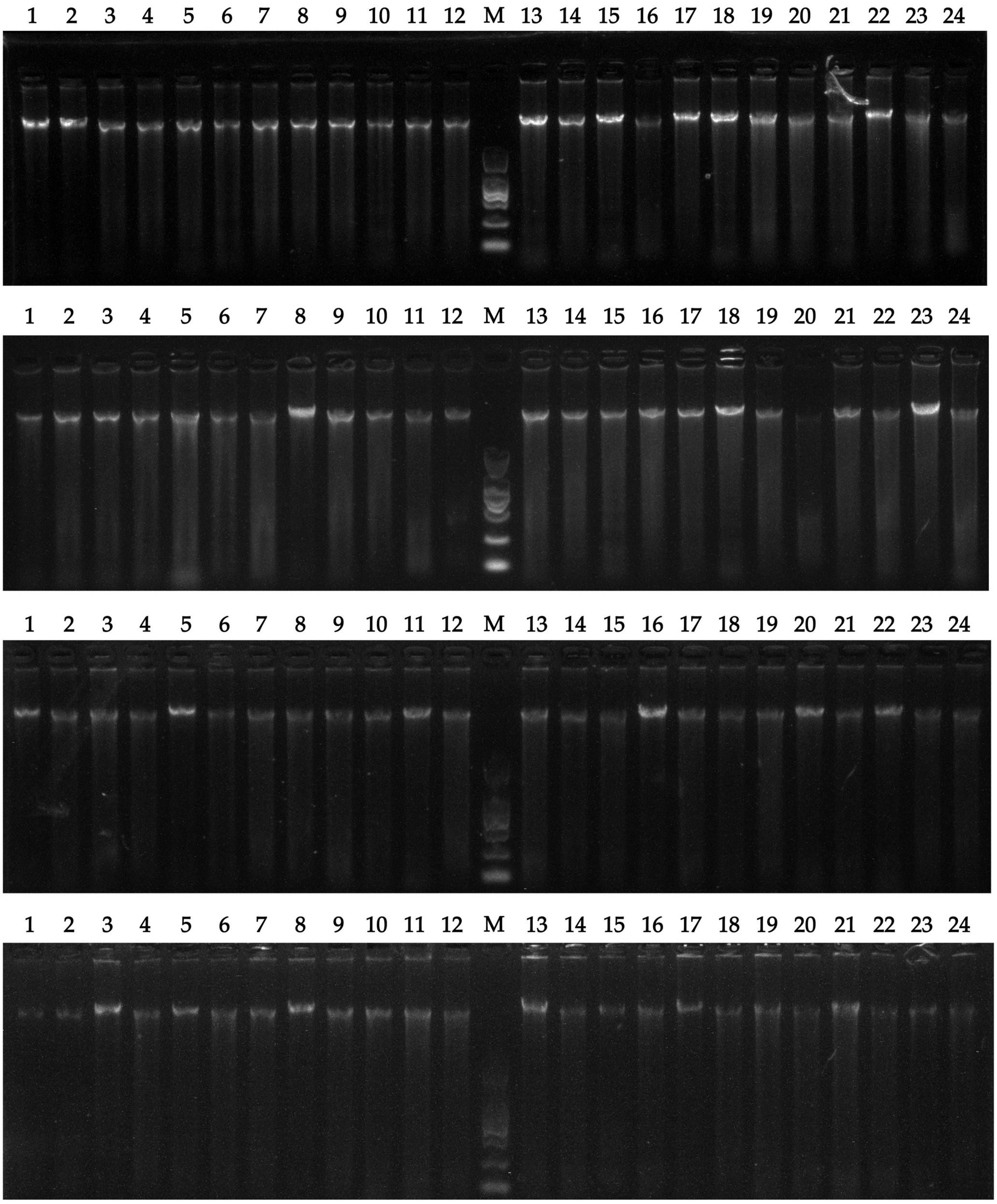

Figure S1. Agarose gel electrophoresis confirms the quality of DNA extracted from 96 materials. Lane M represents the DNA marker DL2000.

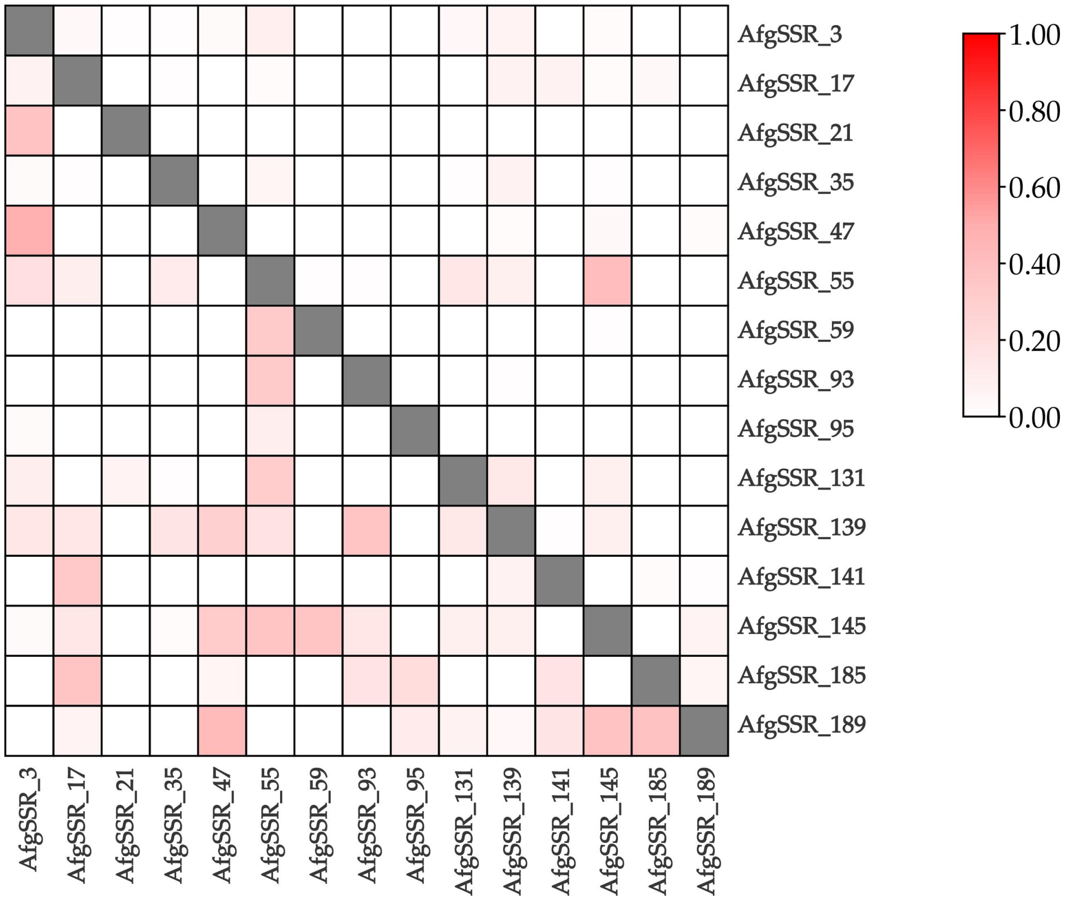

Figure S2. Linkage disequilibrium (LD) among 15 loci. Values in the upper and lower triangles represent R^2 and D' measures, respectively.
